# Reconfiguring the Challenge of Biological Complexity as a Resource for Biodesign

**DOI:** 10.1128/msphere.00547-22

**Published:** 2022-12-06

**Authors:** Erika Amethyst Szymanski, James Henriksen

**Affiliations:** a Department of English, Colorado State University, Fort Collins, Colorado, USA; b Natural Resource Ecology Laboratory, Colorado State University, Fort Collins, Colorado, USA; Lawrence Berkeley National Laboratory

**Keywords:** engineering biology, metaphors, microbial consortia, synthetic biology

## Abstract

Biological complexity is widely seen as the central, intractable challenge of engineering biology. Yet this challenge has been constructed through the field’s dominant metaphors. Alternative ways of thinking—latent in progressive experimental approaches, but rarely articulated as such—could instead position complexity as engineering biology’s greatest resource. We outline how assumptions about engineered microorganisms have been built into the field, carried by entrenched metaphors, even as contemporary methods move beyond them. We suggest that alternative metaphors would better align engineering biology’s conceptual infrastructure with the field’s move away from conventionally engineering-inspired methods toward biology-centric ones. Innovating new conceptual frameworks would also enable better aligning scientific work with higher-level conversations about that work. Such innovation—thinking about how engineering microbes might be more like user-centered design than like programming a computer or building a car—could highlight complexity as a resource to leverage, not a problem to erase or negate.

## PERSPECTIVE

Engineering biology is suffering from a lack of imagination, at least in the language used to conduct it. From its inception, biological complexity has been engineering biology’s central challenge (1). Biological systems involve seemingly uncountable, highly interconnected components, many of which remain poorly described and which are not necessarily fully decomposable (2). Engineering biology involves abstracting these complex systems into machinelike, discrete, interchangeable parts (3). Yet projects are routinely stymied when biological parts interact with context, affecting function in unplanned ways (4, 5)—in other words, when organismal behavior exceeds the machine analogy.

Recent experimental developments have diverged from these foundational (nonliving) engineering analogies, becoming more host- or organism-centric, adding more biology back into the picture (4, 5). Meanwhile, the conceptual infrastructure for designing and building with biology has lagged behind, largely continuing to construct complexity as an engineering challenge through analogizing living things to cars and computers. We suggest that a different set of metaphors or conceptual tools for imagining what microbes (and other cells) are like might reconfigure complexity as engineering biology’s greatest resource, not its greatest barrier.

Synthetic biology has been built on the back of two analogies: first, that cells are machines operated by genomes; second, that if genomes are information storage molecules written in genetic code, scientists should be able to program cells in the same way that they program computers. The conceptual and physical infrastructure of the field is so tightly linked to these analogies that they have become assumptions embedded in the way things get done.

Host-aware strategies have begun to shift away from a strict application of these analogies, incorporating more biological context into engineering approaches. Some aim to alleviate the metabolic burden of genetic constructs by decoupling heterologous gene expression from autochthonous processes (6); others fine-tune engineered pathways in light of a larger picture of resource flux within the cell (7). Yet while some contextual variables can be quantified and modeled, all but the most deliberately constructed biological systems involve far too many potentially relevant parts and connections to comprehensively characterize. The residual undifferentiated details are routinely glossed as “complexity,” especially when they interrupt or impede design goals.

Because of complexity, rational approaches to biodesign become resource-intensive cycles of tinkering with a genetic assembly until it works. The frequent disconnect between the predicted and observed functions of DNA designs has led some to say that engineering biology is not “really engineering,” on the basis of a limited vision of engineering that has permeated much of the field (8–10). That disconnect has also been blamed for why bioengineering chronically lags behind the developmental trajectory anticipated for it on the basis of cognate trajectories of engineering computers. Decoupling biological parts into (hopefully) standardized modules has enabled relatively simple designs that intersect with a cell’s dense, recursive regulatory networks at a limited number of points. However, as design become more ambitious, so too does the likelihood of encounters with complexity exceeding the practical capacity to tinker—even when orthogonal control systems are designed to avoid interfering with cellular processes (3, 10, 11). Moreover, high-level descriptions in inherited engineering terms hide tinkering under the umbrella of the ubiquitous design-build-test (DBTL) cycle, feeding unrealistic expectations about what is scientifically plausible.

A promising bottom-up approach to addressing the challenge of complexity involves building synthetic living systems from well-described parts, limiting the number of parts in the system to the minimum needed to achieve a specific function (12, 13). An alternative, top-down approach involves constructing minimal cells by beginning with existing organisms and then eliminating genetic (and perhaps other) elements found to be unnecessary under specific conditions. A third strategy involves tailoring parts-based assemblies to account for cellular conditions, using modeling or directed evolution.

A limitation of all three approaches is that they are likely to construct cells adept at doing one thing under narrowly defined conditions (12, 14); in contrast, precisely because they are complex and thus responsive and adaptable, microbes and mammalian cells are adept at doing many things under shifting conditions. A second limitation is that they are difficult to achieve. The comprehensive whole-cell model that would ideally anchor the third strategy, in particular, remains out of reach; simplifications must be made. Again, because of complexity, identifying what can be safely eliminated from a model—or a cell—without disabling its utility, disrupting essential cell functions, or causing other genes to become essential or deleterious in turn is tricky. A third difficulty is that, as minimal genome projects have illustrated, a large number of genes are required to sustain life, even under highly controlled conditions, but for unidentified reasons.

Some organism-centric approaches, such as directed evolution strategies, employ complex cellular responsiveness as a design strategy rather than an interruption to design—delegating cycles of trial and error to evolution or, we could say, to complex cell-environment interactions. Our proposition is that engineering biology would benefit from building on this opening by making the tension between the field’s foundational machine analogies and contemporary organism-centric approaches explicit and by innovating alternatives. Experimenting with metaphors that do not reproduce the assumption that cells look or should look like computers and cars, we suggest, is likely to invite additional strategies for employing complexity as a resource rather than a problem to be overcome.

Synthetic biology’s central analogies—expressed in such ubiquitous conceptual infrastructure as the DBTL cycle—configure biological systems as *imperfect* machines because their complexity gets in the way of predictable sequence-function modularity. What would happen if, instead of imagining cells as imperfect machines and trying to make them simpler, engineering biology involved imagining cells as being really good cells? Multipart, redundant, recursive, interacting functional systems enable cells to grow, reproduce, and maintain tightly regulated, finely tuned responses to environmental change. Their responsive and self-amplifying capacities are a major part of why biological systems are useful technologies in the first place. Engineering biology is exciting precisely because machine analogies are imperfect. Cells are imperfect machines, but life is great at being alive.

Machine analogies limit the range of conceivable biodesign strategies by embedding several assumptions: (i) that biological systems should be made increasingly passive and controllable, (ii) that unplanned biological responses constitute undesirable interference in design, and (iii) that engineering biology is lagging behind conventional engineering along an established trajectory for how the field is supposed to develop. Because these assumptions are carried along with “dead” analogies—analogies easily employed without recognizing them as analogies—they become less visible as choices that could be made differently. Their influence remains visible even, for example, in justifications of directed evolution as a stopgap measure en route to better rational design.

Alternative analogies might enable engineering biology to better leverage what could be called cellular expertise, accounting for and working with their responsiveness rather than trying to engineer it away. Where machine analogies suggest disassembling complex networks into decoupled parts, such that they are better defined but no longer responsive, organism-centric analogies suggest working with complex systems’ capacity to respond to change. This shift builds on extant movements in the field to reconfigure biological complexity as a valuable resource rather than an intractable challenge.

We see such organism-centric perspectives as being grounded in three principles.

### Engineering biology differs from engineering automobiles or semiconductors.

This is so not because living and nonliving systems fundamentally differ but because organisms are not the product of human design practices that make establishing predictive principles for structure-function relationships easy. Biological systems may therefore be said to “know” things that researchers do not, and they may respond to change in ways that researchers would not have anticipated and may not be able to intentionally recapitulate.

### Achieving design goals is more useful than making biology into a particular kind of engineering discipline.

Making biology into an engineering discipline prioritizes increasing control over biological systems and reducing their complexity by decomposing networks into discrete parts. This is a very different goal than trying to design, build, and implement useful biological technologies—the direction in which directed evolution and some other host-aware strategies are refocusing the field. This direction might be better served by building effective working relationships with biological systems, with less focus on control and more focus on outcomes.

### Intervening in biological systems is about communication.

In 1934, the biologist and protocybernetician Jakob von Uexküll suggested that all living things inhabit their own *umwelt* or lifeworld, comprised of the phenomena that an organism can sense and effect (15, 16). Organisms communicate with each other when and only when their respective *umwelten* overlap. Organisms can expand their *umwelten* via what von Uexküll called prostheses, or (broadly defined) technologies; scientists, for example, expand their *umwelten* with DNA sequencing, while bacteria expand their *umwelten* with horizontal gene transfer. We envision engineering biology as being about expanding the overlaps in *umwelten* between scientists and the organisms with which they work, so that they can share overlapping goals and effectively communicate toward achieving them.

To exemplify what such an approach might entail, we reconsider the DBTL cycle as applied to designing microbial consortia. Following the DBTL cycle customarily means imagining and constructing a *d*esign *in silico*, *b*uilding that design from synthetic or extracted and amplified DNA, loading the assembly into a biological system to *t*est its function, and *l*earning from what does and does not work to inform a better *d*esign. In microbial consortium engineering, this process may be repeated at several levels of hierarchy to customize microbial strains that are then assembled into a synthetic community or that are introduced into an existing community.

An organism-centric frame that explicitly accounts for microbial responsiveness might reconfigure the DBTL cycle as the listen-parse-respond (LPR) cycle: *l*isten to the microbe or microbial consortia, *p*arse relationships among microbial communications and researcher goals, and *r*espond with an informed intervention to continue the conversation. Genetic material has been analogized to encrypted human or encoded computer languages to make identifying and interpreting genetic “words” analogous to making sense of human language use and to develop new techniques through that analogy (17–19). We extend this analogy by imagining genetic statements as dialogue or discursive resources that microbes use to negotiate environments and that microbes and humans can use to communicate with each other. Consequently, LPR workflows might resemble other negotiated communication scenarios and might encourage more diverse “conversational” strategies in organism-centric experiments. Microbial consortium design might, for example, be described in terms of the following.

### User-centered (participatory) design.

Researchers configure design goals in terms of a problem that can be shared with the microbial community required to enact it, such as how a microbial community with particular characteristics can thrive under particular conditions, and then invite (microbial) users to participate in the design of a solution to the problem. Directed evolution experiments can be seen as participatory design experiments in which scientists equip microbes with a technology (one or more novel genetic statements), ask microbes to use that technology to solve a design problem in the form of a challenging environment, parse the responses of the most successful, and respond with an additional challenge that advances toward a functional design that becomes, effectively, a shared goal (see, e.g., references 20–22).

### Marketing.

Researchers aim to convince a microbial community to adopt specific practices with novel (genetic) resources. To do so persuasively, they need to evaluate and account for microbial responses regarding the product, place (context), price (metabolic cost), and promotion (delivery and incentive to retain and continue using the genetic construct) (see, e.g., reference 4). Reconfiguring typical experimental parameters through this frame may enable making more deliberate and varied use of microbial responsiveness as valuable data rather than a barrier to enacting a design.

### Public engagement.

Researchers aim to dialogue and negotiate with microbial stakeholders to identify a communally acceptable route toward a technoscientific aim. Through this frame, crafting enrichment culture conditions could be seen as a parallel to providing public spaces for mutually beneficial activities.

These ideas may sound dangerously anthropomorphic, but we could just as easily say that engineering metaphors are dangerously mechanomorphic. Scientific reasoning is intrinsically analogical, because to apprehend and make sense of as-yet-unknown phenomena, we must have some idea of what they are *like*. Metaphors such as the DBTL cycle structurally embed analogical reasoning in language, such that doing science without metaphors is impossible (19). Problems therefore arise not because a metaphor is employed, but because the metaphor and the assumptions it carries may be unhelpful for a particular purpose and because it becomes invisible as a metaphor that describes some but not all of a phenomenon’s characteristics. Focusing on machinelike capacities may be less useful than focusing on microbes’ responsive organism-like capacities for achieving biodesign goals that involve contextual dependencies. While the necessity of bioengineering standards is often articulated, a diverse set of approaches to biodesign should expand the long-term resilience of the field and the scope of what it can attempt, in contrast to locking all projects into the same underpinning analogy.

Numerous philosophy of biology papers detail how organisms are not machines so that they can describe why engineering biology fails (22, 23). We are far more interested in how engineering biology succeeds. We are, additionally, not concerned with the ethics of whether organisms *should* be analogized to machines. Instead, we are interested in how to develop successful coworking strategies with organism technologies in light of their distinctive capacities. Leveraging responsiveness and complexity requires rethinking the analogies that underpin biodesign. Doing so will not be a panacea for biodesign challenges. However, reexamining institutionalized assumptions through alternative paradigms may prompt new design strategies in a clogged space.

Biotechnology’s most practical and versatile successes are arguably furthest from the idealized conception of “real” engineering, from recent successes with directed evolution to dynamic self-adapting microbial communities that power wastewater treatment plants (24), spontaneous sourdough bread ferments (25), and microbial ecosystem contributions to sustainable agriculture (26). Biological complexity is a strength in these applications in that microbes—individually and communally—resiliently adapt to changing circumstances while maintaining a functional identity. As Wei and Endy have argued in describing where modularity fails in constructing living systems from nonliving parts, researchers (re)make systems in the image of what they expect them to be (2). Given the diversity of technical approaches now available, engineering biologists have choices: to erase complexity to make cells stupider, or to develop strategies to work with their intelligent complexity (27–29). We think that the latter is, at the very least, equally promising.
